# Gonorrhea and Some of Its Results

**Published:** 1906-02

**Authors:** E. G. Ballenger

**Affiliations:** Atanta, Ga.; Lecturer on Genito-Urinary Diseases, Atlanta School of Medicine


### GONORRHEA AND SOME OF ITS RESULTS.*

BY E. G. BALLENGER, M.D., ATLANTA, GA.
Lecturer on Genito-Urinary Diseases, Atlanta School of Medicine.

GENTLEMEN: Before beginning our regular course I desire to present to you a few facts which I have taken some pains to collect, to show you from the start the importance of gonorrhea as a causative factor of many diseases not only in this branch, but all through your medical course and in your practice in after-life. Of course it is natural for every professor and lecturer to think that his own branch is one of the most important. I believe though if you will consider carefully the facts I have collected to show the far-reaching effect of gonorrhea, you will be willing to admit that the time you devote to this subject will not be entirely misspent.

I also will touch briefly upon a few points as to the education of the public, and especially the young men in regard to the dangers and the methods of prophylaxis.

So common is gonorrhea and so mild are many of the cases, that not only the laity, but a large per cent. of physicians and surgeons have developed an indifference that is almost criminal.

Neisser holds that gonorrhea, with perhaps the exception of measles, is the most widespread of all diseases (1). Morrow asserts that it is even a more potent factor of depopulation than syphilis, and that a large per cent. of involuntary childlessness is due to it.

He also thinks the extent to which gonorrhea prevails in private life is much greater than is generally supposed, and makes the startling statement that there is more infection among virtuous wives than in professional prostitutes. The truth of this statement becomes more reconcilable when one considers how much larger is the number of virtuous wives than is the number of prostitutes, and that "powerful motives lead to their concealment." Unfortunately the mildness of many cases gives the patient a false sense of the gravity of his condition, if neglected, and his suffering is rarely sufficient punishment for his sin, until some complication follows,

*Opening lecture at the Atlanta School of Medicine.

or he desires to marry, but can not, because of the uncertainty with which he is harassed, not only on account of the danger of infecting an innocent woman, but also as to his future health and potency. Many of these cases become utterly miserable neurasthenics from the profound mental impression made by the disease affecting the sexual organs, for next to a man's love of life comes that of procreation, and his sexual instinct, which "is given to man for two reasons, to perpetuate the species and to rivet the tie between husband and wife by mutual endearment" (2). Is, then, his mental anguish unnatural?

This is only the history of a certain class; others are apparently cured and of their own accord, or upon the advice of a careless physician, who gives his consent after merely inquiring as to the general symptoms and the discharge, the patient then marries a pure woman "only to deposit in her lap—as a wedding gift from his first wife, the prostitute—the seeds of a foul disease which makes her innocent wifehood a source of pain and misery, and often renders motherhood impossible. The prostitute takes a poetic revenge, and by the disease she gives her paramour forever seals the fountain of nature and restrains her supplanter from acting beyond her own (the prostitute's) sphere, even though she be a thousand times legalized as a wife by church and court" (3).

Is it right to treat so serious a matter as a joke? Should not a young man early in his youth receive a certain amount of education upon such an important subject and one with such disastrous possibilities? Ought this part of the education of a son be left to the servants or his ignorant (and perhaps profligate) companions, who little realize the dangerous pitfalls even if they themselves have fallen therein?

A father can not ease his conscience by thinking his son will not yield to the temptation or will come out unscathed. With the plain fact staring him in the face, that 80 per cent. of men contract gonorrhea, the sophistry of such reasoning is evident. The time to educate is before the disease is contracted, for then if a competent and conscientious surgeon takes charge of him, his one-sided education will be completed, but think what a price to pay! Consider the moral degradation, the physical weakness and the mental changes that have been wrought as chains of iron to handicap his career and perhaps wreck his life. It is not claimed that education will create an Utopia, but fear of impending danger would certainly save many from the maelstrom of venereal diseases if they were only shown the dangers in time. This education in addition to warning as to the peril should also encourage and even urge the young man that continence is not incompatible with perfect health. The double code of moral laws is to be condemned, whereby a man may sin without offending society, and yet the same sin means the woman's ruin. I do not advocate lowering the standard for women, but raising it for men.

Morrow says thousands of young women suffer moral and physical shipwreck from ignorance of the fact that loss of virtue often carries with it the loss of health. "The vast majority of men who carry disease and death into their families do so ignorantly" (4). Undoubtedly many disasters in married life and untold suffering could be averted if the patients were properly advised even after they have contracted the disease. Who is to blame in these cases? Most assuredly the physician, who is almost always insufficiently trained in the methods of determining whether or not a patient can marry with impunity. This should be a standing question in all examinations in surgery, not only in the medical colleges, but also in the State board examinations, and in this way compel the student and physician to be prepared upon this subject. I assert no question could be asked that would be more practical, nor one that will be of more value in preventing sterility, disease, suffering and death.

Gonorrhea is the most amenable of venereal diseases to individual prophylaxis, and yet one hesitates at first to advocate any method which might perhaps increase the evil by lessening the danger which undoubtedly is a great conservator of social purity. When, however, we consider that the social evil has existed through all ages, and that all attempts at suppression have failed, from the rigid laws of Maria Theresa at Vienna and the Popes at Rome (5), down to the present day, and have invariably increased the numbers of the clandestine prostitutes (who is always more dangerous), that control measures have been equally as inefficient as those directed toward suppression, that all history and knowledge of human nature have shown that prostitution can not be suppressed, and as venereal diseases are the worst results, it follows then that efforts must be directed toward protection from these dangers, in behalf of the wife and the State as well as the sinner, if he insists upon exposing himself. Explain to him that there is always danger and urge continence, but also advise him as to prophylactic measures, in case he exposes himself, which, it is sad to relate, will be of frequent occurrence. If he will not reform we are justified in behalf of an innocent wife to protect her and their offspring.

The use of the condon greatly lessens the danger of gonorrhea but it may rupture during coitus. Good results also follow injections of 10 per cent. argyrol after urinating and thoroughly washing the penis. The region of the meatus should be smeared with the same solution to kill any gonococci outside, while if the injection is properly given and retained five minutes the germs in the anterior urethra are destroyed. There is, however, always the danger of syphilis and a chancroid which must be impressed upon the patient.

Other important sociological problems are to be considered in attempting any method preventing venereal diseases; the regulation of sexes in industrial life; the low wages received by working women; the more rigorous attempts to control abortions; and efforts to segregate the prostitutes in certain sections of the cities and leave them there undisturbed in order to lessen the number of the more dangerous clandestines. I regret that it is not within the province of this lecture to enter into the details of these subjects.

To show further the necessity of devoting more time and attention to this widespread though insidious disease that stalks through every nation, causing untold suffering, and making invalids of strong men and women, let us consider briefly some of its various phases:

1. *The Monetary Loss.*—So enormous are the figures and so meager and unreliable are the statistics, that I will ask you to make a mental calculation for yourself and estimate about the average cost of an average patient for necessary expenses incident to the disease, and to that add the loss of time, the loss in business from physical weakness and mental worry, and the loss to the nation from lessened capacity for production. Eighty per cent. is the usual estimate of the men who have gonorrhea (6). According to the U. S. census 1900, there are about 20,000,000 men in these United States between 20 and 50 years of age. Eighty per cent. of this number is 16,000,000. Now multiply this by the average loss of each patient to himself and the State, and the quotient will show part of the monetary loss from gonorrhea alone.

2. *Invalidism and Operations in Women.*—It is under this head that we see the appalling results of gonorrhea. Humiston and Price claim from 90 to 95 per cent. of abdominal operations performed in the world to-day are required on account of infections, adhesions and pus collections due to gonorrhea (7). These are higher than the percentages given by other authorities, but even the lowest is enough to stir the most indolent to activity.

In addition to the operations, equally frequent are those patients who are made invalids for life. Worse than these, however, are the cases who become mentally unbalanced on account of the removal of their sexual organs which were so damaged by the inflammatory process that the gynecologist found them useless or dangerous. A large number of these cases are due to latent gonococci from apparently cured husbands.

3. *Sexual Neurasthenia*, so frequently caused by gonorrhea, is one of the most intractable conditions with which they have to contend, and undoubtedly makes the patient more unhappy than any other non-fatal disease. From the number of cases with which I have come in contact, who have explained how depressed they were, and that on a number of occasions they had contemplated suicide, I feel justified in saying that this condition next to financial embarrassment and love affairs is the main cause of self-destruction. I am equally positive that a certain per cent of these cases become insane. It is also without doubt a frequent cause of divorce.

4. *Sterility.*—Through its effect on the epididymes and prostate gland it plays the main role in causing sterility and impotence in men. Benzler (8) followed the history of 474 soldiers with gonorrhea and found sterility resulted in 10 per cent. of the cases of urethral involvement; 23 per cent. where there was unilateral epididymitis and 41 per cent. where the epididymitis was double. Impotence is one of the disastrous results of damage to the prostatic urethra by the inflammation. It may be complete or partial, as imperfect erection, premature ejaculation, or loss of normal sensation apparently due to the destruction of the nerve endings in this part of the urethral canal (9).

According to Neisser, 50 per cent. of all involuntary childless marriages are due to gonorrhea. Cervical catarrh prevents the entrance of the spermatozoa, thereby rendering the woman sterile. After the inflammatory process has reached the endometrium and tubes, still further obstruction prevents the fertilization of the ovum even when the disease has not progressed to pus formation.

Bland-Sutton says gonorrhea is the chief cause of sterility of the prostitute class (7). Morrow has shown that women having it frequently can only give birth to one child, and subsequent trouble is likely to date from this event which furnishes ideal conditions for the growth and spread of the gonococci.

Some of the worst results of gonorrhea are not due so much to gonococci, which as a rule limit themselves to the mucous membranes, but to the secondary infections that are afforded extremely favorable conditions for their growth.

DeWarker (10) gives the first place to gonorrhea in the three diseases (gonorrhea, tuberculosis and syphilis) that press urgently for solution. He gives it the first place, not because it is more destructive to life than tuberculosis, but on account of its greater dangers to society, in its power to impair fertility and to permanently infringe upon usefulness.

5. *General Gonococcal Infections* have also been reported very frequently in recent years, and Winn (11) states that the lesions and symptoms depend upon three factors:

(1) Direct infection with the gonococcus itself; (2) absorption of a toxin (gonotoxin), and (3) mixed infection with other germs. Krause (2) demonstrated gonococci in the blood during life, and Ullmann has reported five cases of fatal gonorrheal pyemia, in four of which a necropsy showed the point of origin to be a prostatic abscess. Gonorrheal infection has no special clinical features which can differentiate it from other pyemias, and a positive diagnosis can only be made by a bacteriological examination of the blood or from an accessible lesion. Krause calls attention to the ease with which gonococci may be overlooked, which only grow to small colonies in blood, and in cultivating them the agar must not be heated above 41 degrees C.

6. *Blindness.*—Formerly about 50 per cent. of all blindness was due to gonorrhea, but since the adoption of Crede's prophylactic measures this has been reduced to about 20 per cent. Even with this reduction it may still be given as evidence against gonococci, and to further strengthen the case against their attack on the innocent helpless children Holt has recently shown that gonococcal infections and vaginitis are exceedingly frequent in institutions for children, highly contagious and difficult to control. He says where this infection is known it is justly regarded as the *bete noire* of institutions for children, the importance of which has only just begun to be appreciated. Twenty-six cases of gonococcal arthritis are reported, and it is claimed that a pyemic arthritis in a young infant is much more frequently due to the gonococcus than to the streptococcus or any other pyogenic organism.

7. *The Need for Reporting These Diseases.*—All efforts to study venereal diseases are hampered by the absolute lack of any reliable statistics in this country, and the attempt to have them reported purely for statistical purposes, without the names of the individuals, is most heartily approved. "While venereal diseases cause more deaths and deplete the population by causing abortion and preventing conception more than any other known disease or condition, this subject is largely ignored by sanitary bureaus in their registration of contagious diseases" (7). It is only in this way that the laity, physicians, and especially philanthropists, are to be shown the necessity of taking steps to check the ravages of these diseases which, although sapping the vitality and life-blood of the nation, continue to be ignored. No city in this country has adequate hospital accommodations for the venereal patients. There is no more crying need to-day than for efficient treatment and medicine for all poor patients before the disease has tightened its relentless hold, and they are doomed, for life, to be sterile, impotent, or invalids.

In conclusion, gentlemen, I hope the facts I have reviewed will enable you to appreciate more fully the importance of gonorrhea, its widespread and far-reaching effect, and trust none of you will admit it a disease to be feared no "worse than a cold in the head," and will combat any such criminal ideas on all occasions.

BIBLIOGRAPHY.

1. Holton: *Journal American Medical Association*, March 11, 1905.
2. Peterkin: *North West Med.*, Vol. II, No. 9, 1905.
3. Kelly: *Jour. A. M. A.*, March 4, 1905.
4. Heidingsfeld: *Jour. A. M. A.*, January 30, 1904.
5. Weiss: *Jour. A. M. A.*, January 24, 1903.
7. Johnson: *Jour. A. M. A.*, March 11, 1905.
8. Benzler: *Archiv f. Dermatol, and Syph.*, Band XLV, Heft 1.
9. *Progres. Med.*, December, 1899.
10. De Warker: *New York and Philadelphia Med. Journal.*
11. Winn: *The Lancet*, February 11, 1905.
12. Krause: *Berlin klin. Woch.*, May 9, 1904.

---

### FIFTEENTH ANNUAL MEDICAL CONGRESS.

Interest is increasing in the approaching session of the International Medical Congress, which is to be held in Lisbon, April 19 to 26. The preliminary program and itinerary of the American party which is being organized, describes a most interesting trip at very low cost.

Dr. John Musser, of Philadelphia, is chairman of the National Committee, and Dr. Ramon Guiteras, 75 West 55th street, New York City, is the secretary, to whom all applications for membership in the Congress and communications regarding papers should be addressed.

The sailing date of the American party is April 7, by the North German Lloyd Steamship, Koenig Albert.

The arrangements are in the hands of Dr. Chas. Wood Fassett, of St. Joseph, to whom applications for reservations should be made. In order that proper hotel accommodations may be secured in Lisbon, there should be no delay on the part of those who contemplete attending the Congress.

